# Mapping the Fungal Battlefield: Using *in situ* Chemistry and Deletion Mutants to Monitor Interspecific Chemical Interactions Between Fungi

**DOI:** 10.3389/fmicb.2019.00285

**Published:** 2019-02-19

**Authors:** Sonja L. Knowles, Huzefa A. Raja, Allison J. Wright, Ann Marie L. Lee, Lindsay K. Caesar, Nadja B. Cech, Matthew E. Mead, Jacob L. Steenwyk, Laure N. A. Ries, Gustavo H. Goldman, Antonis Rokas, Nicholas H. Oberlies

**Affiliations:** ^1^Department of Chemistry and Biochemistry, University of North Carolina at Greensboro, Greensboro, NC, United States; ^2^Department of Biological Sciences, Vanderbilt University, Nashville, TN, United States; ^3^Faculdade de Ciências Farmacêuticas de Ribeirão Preto, Universidade de São Paulo, São Paulo, Brazil

**Keywords:** co-culturing, *laeA*, *in situ*, fungi, secondary metabolites, interspecific interactions

## Abstract

Fungi grow in competitive environments, and to cope, they have evolved strategies, such as the ability to produce a wide range of secondary metabolites. This begs two related questions. First, how do secondary metabolites influence fungal ecology and interspecific interactions? Second, can these interspecific interactions provide a way to “see” how fungi respond, chemically, within a competitive environment? To evaluate these, and to gain insight into the secondary metabolic arsenal fungi possess, we co-cultured *Aspergillus fischeri*, a genetically tractable fungus that produces a suite of mycotoxins, with *Xylaria cubensis*, a fungus that produces the fungistatic compound and FDA-approved drug, griseofulvin. To monitor and characterize fungal chemistry *in situ*, we used the droplet-liquid microjunction-surface sampling probe (droplet probe). The droplet probe makes a microextraction at defined locations on the surface of the co-culture, followed by analysis of the secondary metabolite profile via liquid chromatography-mass spectrometry. Using this, we mapped and compared the spatial profiles of secondary metabolites from both fungi in monoculture versus co-culture. *X. cubensis* predominantly biosynthesized griseofulvin and dechlorogriseofulvin in monoculture. In contrast, under co-culture conditions a deadlock was formed between the two fungi, and *X. cubensis* biosynthesized the same two secondary metabolites, along with dechloro-5′-hydroxygriseofulvin and 5′-hydroxygriseofulvin, all of which have fungistatic properties, as well as mycotoxins like cytochalasin D and cytochalasin C. In contrast, in co-culture, *A. fischeri* increased the production of the mycotoxins fumitremorgin B and verruculogen, but otherwise remained unchanged relative to its monoculture. To evaluate that secondary metabolites play an important role in defense and territory establishment, we co-cultured *A. fischeri* lacking the master regulator of secondary metabolism *laeA* with *X. cubensis*. We found that the reduced secondary metabolite biosynthesis of the Δ*laeA* strain of *A. fischeri* eliminated the organism’s ability to compete in co-culture and led to its displacement by *X. cubensis*. These results demonstrate the potential of *in situ* chemical analysis and deletion mutant approaches for shedding light on the ecological roles of secondary metabolites and how they influence fungal ecological strategies; co-culturing may also stimulate the biosynthesis of secondary metabolites that are not produced in monoculture in the laboratory.

## Introduction

Fungi naturally grow in competitive environments, such as soil, plants, and animal tissues (Bertrand et al., 2013a). They have evolved a diversity of ecological strategies to combat their competitors, which include rapid growth, stress recovery, and the use and negation of inhibitors (Losada et al., 2009). Interaction-driven secondary metabolite discovery (i.e., co-culturing) can exploit the ability of fungi to produce secondary metabolites that have evolved to combat various competitors. Co-culturing experiments often activate the biosynthesis of defense secondary metabolites, allowing one to “see” how fungi respond, chemically, within a competitive environment (Fox and Howlett, 2008; Kuhar et al., 2015). They also provide a window to understanding fungal ecology and the ecological relevance of secondary metabolites.

Previous studies have shown that fungal genomes have a rich diversity of biosynthetic gene clusters (Pel et al., 2007; Helaly et al., 2018; Keller, 2018; Rokas et al., 2018), and that fungi have the potential to produce numerous chemically diverse secondary metabolites; however, a large proportion of biosynthetic gene clusters are silent under traditional laboratory culture conditions (Keller et al., 2005; Gross, 2007; Challis, 2008; Scherlach and Hertweck, 2009; Grigoriev et al., 2013; Keller, 2018). One possibility is that only a fraction of the potential secondary metabolites are produced, likely due to fungal domestication as a result of repeated culturing at optimal media conditions. Another possibility is that the stressors that elicit the expression of certain secondary metabolites are not present (Keller, 2018; Xu et al., 2018).

Co-culturing is a promising approach for activating regulatory mechanisms that result in the biosynthesis of otherwise silenced secondary metabolites for targeted interaction discovery (Huang et al., 2014). For example, when *Trichophyton rubrum* and *Bionectria ochroleuca* were co-cultured, there was an activation of silent biosynthetic gene clusters, as evidenced by a new secondary metabolite that was only present in the zone separating the two fungi (Bertrand et al., 2013b). It has also been shown that co-culturing two fungi can increase the quantity of certain secondary metabolites that were previously being biosynthesized (Nonaka et al., 2011; Bertrand et al., 2014; Xu et al., 2018). A potential reason for these activations is interspecific interactions. These interactions can cause chemical cues to be exuded to the environment between two or more species competing for nutrition and space that could result in the activation of biosynthetic gene clusters (Luo et al., 2017; Mandelare et al., 2018; Wang W. et al., 2018).

Over the last decade numerous mass spectrometry tools have been utilized to study microbial interactions *in situ* to obtain a holistic understanding of the chemical interactions between two competing microbes (Sica et al., 2016c; Eckelmann et al., 2018; González-Menéndez et al., 2018). Most studies have explored bacterial-bacterial interactions and fungal-bacterial interactions *in situ* in a Petri plate (Wang et al., 2015; Michelsen et al., 2016; Eckelmann et al., 2018; González-Menéndez et al., 2018), but there are a few studies that have explored the mapping and characterization of fungal-fungal interactions *in situ* (Tata et al., 2015; Sica et al., 2016c; Bai et al., 2018; Chen et al., 2018). One of the main issues that precludes *in situ* studies on fungi is the uneven morphology (heterogeneous topography) of fungal mycelia, which is not easily amenable to *in situ* ionization techniques without modifying the culture (Bertrand et al., 2013a; Sica et al., 2014, 2015, 2016c). The problem of uneven topology can be overcome by using the droplet–liquid microjunction–surface sampling probe (droplet probe) (Kertesz and Van Berkel, 2010, 2013). Importantly, mapping the chemical entities of fungi as they compete *in situ* can address chemical ecology questions, such as where (spatially) and when (temporally) the compounds are biosynthesized, and such data are typically lost in previous studies on co-culturing fungi through the use of a traditional chemical extraction processes (Wakefield et al., 2017; Abdelwahab et al., 2018; Mandelare et al., 2018).

A connection has been established in the literature between the production of secondary metabolites produced by fungi and their role in ecological chemical interactions (Kusari et al., 2012; Spiteller, 2015). It is well known that *laeA* (loss of *aflR* expression, with *aflR* being the regulatory gene for aflatoxin biosynthesis) is a global regulator and controls both fungal growth and development and is responsible for over 50% of secondary metabolites produced in *Aspergillus* as well as other genera (Sarikaya-Bayram et al., 2015; Lind et al., 2016). It has been reported that deletion mutants *laeA* are less pathogenic (Sarikaya-Bayram et al., 2015). However, what is not known is what would happen if a *laeA* mutant of *Aspergillus fischeri* were grown in co-culture with a competing fungus. In other words, by monitoring the chemical interaction between fungi *in situ*, could a wild type and *laeA* mutant of *Aspergillus fischeri* co-cultured with another fungus help answer the role of secondary metabolites for defense? We hypothesized, based on the large body of previous literature, that since *laeA* is integral to both growth and development as well as production of secondary metabolites (Sarikaya-Bayram et al., 2015), a Δ*laeA* mutant would be unable to battle with other fungi. Thus, as the chemical arsenal of the Δ*laeA* mutant is affected, this in turn would affect its ability to occupy space and survive. Such experimental studies would help shed light on the biological roles of secondary metabolites in fungi and provide experimental evidence for the ecological role of secondary metabolites in fungi *in situ*.

In this study, we evaluated the co-culturing of two fungi, *Aspergillus fischeri* and *Xylaria cubensis, in situ* and observed distinct chemical profile changes resulting from the interspecific interactions between them. *X. cubensis* commonly occurs as an endophyte (endosymbiotic) and decomposer (saprobic) (Helaly et al., 2018) and biosynthesizes the fungistatic secondary metabolite griseofulvin, an FDA-approved drug (Sica et al., 2016c; Paguigan et al., 2017). Fungistatic denotes that it inhibits fungal growth, rather than kills competing fungi, and we hypothesized, therefore, that the resulting stress in a co-culture environment would allow us to examine how secondary metabolite production changes when the two species interact.

*Aspergillus fischeri* was chosen due to its genetic tractability and its evolutionary relatedness with *A. fumigatus*, the human pathogen (Samson et al., 2007; Brosch et al., 2008; Mead et al., 2019). It also has its own metabolite weaponry in the form of mycotoxins (Mead et al., 2019). The secondary metabolites of *Aspergilli*, including *A. fischeri*, are largely controlled by the master regulator protein *laeA* (Bok and Keller, 2004; Lind et al., 2016; Lv et al., 2018; Mead et al., 2019; Wang B. et al., 2018). A deletion strain of *A. fischeri* where *laeA* was knocked out (Δ*laeA*) was co-cultured with *X. cubensis*. Concomitant with reduction of the biosynthesis of secondary metabolites, *A. fischeri* lost its competitive advantage, and *X. cubensis* was able to out compete it, as compared to the co-culture with wild type *A. fischeri*.

## Materials and Methods

### General Experimental Procedures

The NMR data were collected using a JOEL ECS-400 spectrometer, which was equipped with a JOEL normal geometry broadband Royal probe, and a 24-slot autosampler, and operated at 400 MHz for ^1^H and 100 MHz for ^13^C, a JOEL ECA-500 spectrometer operating at 500 MHz for ^1^H and 125 MHz for ^13^C (Both from JOEL USA, Inc.), or an Agilent 700 MHz spectrometer (Agilent Technologies), equipped with a cryoprobe, operating at 700 MHz for ^1^H and 175 MHz for ^13^C. HRMS experiments utilized either a Thermo LTQ Orbitrap XL mass spectrometer or a Thermo Q Exactive Plus (Thermo Fisher Scientific); both were equipped with an electrospray ionization source. A Waters Acquity UPLC (Waters Corp.) was utilized for both mass spectrometers, using a BEH C_18_ column (1.7 μm; 50 mm × 2.1 mm) set to a temperature of 40°C and a flow rate of 0.3 ml/min. The mobile phase consisted of a linear gradient of CH_3_CN-H_2_O (both acidified with 0.1% formic acid), starting at 15% CH_3_CN and increasing linearly to 100% CH_3_CN over 8 min, with a 1.5 min hold before returning to the starting condition. The HPLC separations were performed with Atlantis T3 C_18_ semi-preparative (5 μm; 10 × 250 mm) and preparative (5 μm; 19 × 250 mm) columns, at a flow rate of 4.6 ml/min and 16.9 ml/min, respectively, with a Varian Prostar HPLC system equipped with a Prostar 210 pumps and a Prostar 335 photodiode array detector (PDA), with the collection and analysis of data using Galaxie Chromatography Workstation software. Flash chromatography was performed on a Teledyne ISCO Combiflash Rf 200 and monitored by both ELSD and PDA detectors.

### Isolation and Identification of *Aspergillus fischeri* and *Xylaria cubensis*

*Aspergillus fischeri* strain NRRL 181 was obtained from ARS Culture Collection (NRRL) (Mead et al., 2019). The *A. fischeri ΔlaeA* mutant was prepared using methods outlined previously (Mead et al., 2019). *Xylaria cubensis* strain G536 was isolated as an endophyte from surface sterilized twigs of *Asimina triloba* and identified using molecular methods as outlined previously (Sica et al., 2016c).

### Cultures of *Aspergillus fischeri* and *Xylaria cubensis* on Solid Nutrient Media in Petri Plates

**FIGURE 1:**
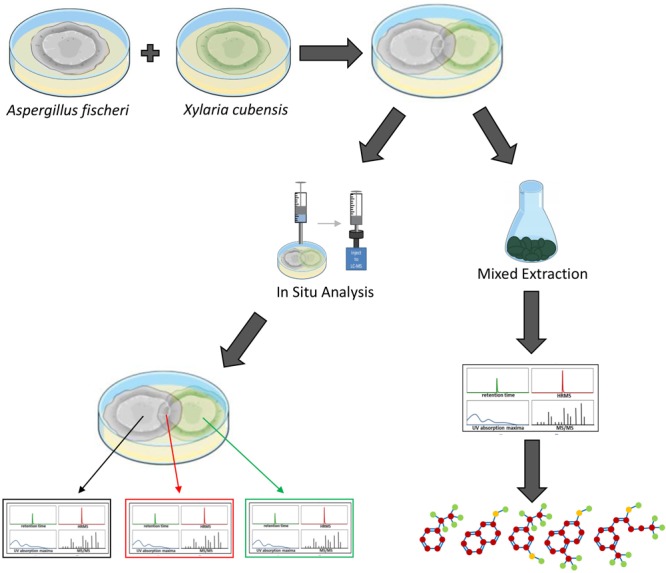
Overview of how the secondary metabolites biosynthesized during the monoculture and co-culture were analyzed. *Aspergillus fischeri* (NRRL 181) and *Xylaria cubensis* (G536) were grown as monocultures in separate Petri plates before they were co-cultured. The co-culture and monocultures are then analyzed (left) via *in situ* microextractions where spatial mapping was performed, which provides a map of how the secondary metabolites are distributed along the co-culture landscape in the Petri plate. Large-scale fermentation (mixed culture; right) where both fungi were transferred together onto solid fermentation media in 250 mL Erlenmeyer flasks and the co-culture was allowed to grow for 3–4 weeks. Subsequently the cultures were extracted with organic solvents using standard natural product extraction and characterization protocols.

*Aspergillus fischeri* and *Xylaria cubensis* were maintained on potato dextrose agar (PDA; Difco). To establish individual monocultures, an agar plug from the leading edge of the colony was cut out aseptically and transferred onto 50 mm Petri plates with oatmeal agar (OMA; Difco). Oatmeal was chosen since this condition yielded enhanced biosynthesis of secondary metabolites (Mead et al., 2019). The monocultures were grown for 18 days. For co-culture, an agar plug from each of the two strains was placed approximately 40 mm apart on the Petri plate with OMA media. The Petri plates were incubated at room temperature (∼22°C) for 18 days under 12-h light/dark cycles. For both monoculture and co-culture growths, *in situ* analyses were conducted after 18 days (Figure 1).

### *In situ* Chemical Analysis Using Droplet Probe

**FIGURE 2:**
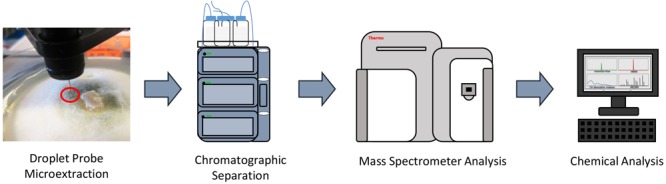
General procedure for how the droplet probe works. A microextraction using 1:1 H_2_O:MeOH (H_2_O for droplet retention and MeOH for extraction) is performed *in situ* at the desired location on the surface of the living fungal cultures. That droplet is then injected into an LC-MS system, which allows for separating and analyzing the different peaks, which correspond to different secondary metabolites.

To characterize the secondary metabolic profiles of monocultures and co-cultures *in situ*, the droplet probe was used to chemically map the locations of the biosynthesized secondary metabolites. Sampling the surface of fungi *in situ* was performed using the droplet-liquid microjunction-surface sampling probe (droplet probe), using methodology detailed previously (Kertesz and Van Berkel, 2010, 2013; Sica et al., 2015, 2016a,c). Briefly, it uses a CTC/LEAP HTC PAL auto-sampler (LEAP Technologies Inc.) that has been converted to an automated droplet probe system. The microextractions (∼5 μl) were performed using 1:1 MeOH:H_2_O. The droplet was dispensed at a rate of 2 μl/s from the needle, held on the fungal surface for 2 s, and then withdrawn at the same rate before injecting into an LC-MS system (Figure 2). The LC-MS used a solvent system of CH_3_CN and H_2_O, with both being acidified with 0.1% formic acid. The chromatography method had a flow rate of 0.3 ml/min and a gradient of 15–100% CH_3_CN over 8 min, holding at 100% for 2 min, and returned to the starting conditions for 2 min. The surface of the fungal mycelia were sampled for secondary metabolites.

*Xylaria cubensis* and wild type *Aspergillus fischeri* were grown side by side on OMA for 14 days. This plate was sampled spatially perpendicular to the interface of the co-culture at eight locations. Wild type *A. fischeri* was sampled in three locations. The first sampling point was the point of inoculation, and then two points were evenly spaced before the junction of the two fungal mycelia. Two spots were sampled within the junction where both fungal mycelia overlap. Two spots were sampled in the mycelium of *X. cubensis* with them being evenly distributed through the mycelium. The final location sampled was the stroma of *Xylaria cubensis* (Supplementary Figure S3). This was performed on three separate Petri plates for biological replication, and the results were similar.

### Large-Scale Fermentation of *Aspergillus fischeri* and *Xylaria cubensis* Co-cultures

To identify the secondary metabolites that were biosynthesized during the co-culture experiment, they were grown in large-scale fermentation to isolate and characterize the secondary metabolites. To inoculate oatmeal cultures, agar plugs from *A. fischeri* and *X. cubensis* growths on PDA were cut from the edge of the cultures and transferred to separate liquid seed media that contained 10 ml YESD broth (2% soy peptone, 2% dextrose, and 1% yeast extract; 5 g of yeast extract, 10 g of soy peptone, and 10 g of D-glucose in 500 ml of deionized H_2_O) and cultivated at 22°C with agitation at 100 rpm for 3 (*A. fischeri*) and 5 days (*X. cubensis*). YESD seed cultures of both fungi grown individually were subsequently used to inoculate 16, 250 ml Erlenmeyer flasks that contained 10 g of autoclaved Quaker Breakfast Oatmeal each (10 g of oatmeal with 17 ml of deionized H_2_O and sterilized for 15–20 min at 121°C) and grown at room temperature for 4 weeks.

### Chemical Characterization of *Aspergillus fischeri* and *Xylaria cubensis* Co-cultures

To characterize the secondary metabolites produced from the co-culture, the fungal cultures underwent extraction and purification to isolate secondary metabolites. The large-scale co-culture was extracted by adding 60 ml of (1:1) MeOH–CHCl_3_ to each 250 ml flask, chopping thoroughly with a spatula, and shaking overnight (∼ 16 h) at ∼ 100 rpm at 22°C. The culture was filtered *in vacuo*, and 90 ml CHCl_3_ and 150 ml H_2_O were added to the filtrate. The mixture was stirred for 30 min and then transferred to a separatory funnel. The organic layer (CHCl_3_) was drawn off and evaporated to dryness *in vacuo*. The dried organic layer was reconstituted in 100 ml of (1:1) MeOH–CH_3_CN and 100 ml of hexanes, transferred to a separatory funnel, and shaken vigorously. The defatted organic layer (MeOH–CH_3_CN) was evaporated to dryness *in vacuo*.

The defatted extract was dissolved in CHCl_3_, absorbed onto Celite 545 (Acros Organics), and fractionated by normal phase flash chromatography using a gradient of hexane-CHCl_3_-MeOH at a 30 ml/min flow rate and 61.0 column volumes, which yielded five fractions. Fraction 1 was purified further via preparative HPLC using a gradient system 90:10 to 100:0 of CH_3_CN-H_2_O with 0.1% formic acid over 30 min at a flow rate of 16.9 ml/min to yield eight subfractions. Subfraction eight (32.74 mg), which eluted at 30 min, yielded aszonalenin (**2**) and fumitremorgin A (**4**). Fraction 2 was purified further via preparative HPLC using a gradient system 20:80 to 100:0 of CH_3_CN-H_2_O with 0.1% formic acid over 30 min at a flow rate of 16.9 ml/min to yield twelve subfractions. Subfractions 2, 3, 4, 5, 9, and 10 yielded dechloro-5′-hydroxygriseofulvin (**11**) (0.20 mg), 5′-hydroxygrisoefulvin (**10**) (0.48 mg), dechlorogriseofulvin (**9**) (0.80 mg), griseofulvin (**8**) (0.88 mg), verruculogen (**6**) (0.18 mg), and sartorypyrone A (**1**) (0.34 mg), which eluted at approximately 13.4, 15.2, 15.7, 17.5, 26.1, and 30.3 min, respectively. Fraction 3 was purified further via preparative HPLC using a gradient system 40:60 to 75:25 of CH_3_CN-H_2_O with 0.1% formic acid over 30 min at a flow rate of 16.9 ml/min to yield twelve subfractions. Subfractions 3, 4, 5, 6, 7, 8, and 12 yielded compounds cytochalasin D (**12**) (16.64 mg), acetylaszonalenin (**3**) (5.94 mg), 7-*O*-acetylcytochalasin B (**16**) (0.47 mg), cytochalasin C (**14**) (1.08 mg), hirsutatin A (**17**) (0.76 mg), fumitremorgin B (**5**) (0.41 mg), zygosporin E (**15**), and the C-11 epimer of verruculogen TR-2 (**7**), which eluted at approximately 13.5, 15.6, 18.5, 20.0, 24.0, 27.5, and 30.0 min, respectively. Compounds **15** and **7** co-eluted in a single fraction (18.37 mg) and were further purified via preparative HPLC using a gradient system 50:50 to 55:45 of CH_3_CN-H_2_O with 0.1% formic acid over 30 min at a flow rate of 16.9 ml/min to yield 0.65 and 1.73 mg, respectively.

### LC-MS Analysis

To detect metabolites, LC-MS analysis was conducted in the positive ion mode. The mass spectrometer scanned across a mass range of *m/z* 200 to 2000 at a resolution of 70,000, and a spray voltage of 4,000. It was coupled to an Acquity UPLC system (Waters Corp.), which had a flow rate of 0.3 ml/min and utilized a BEH C_18_ column (2.1 mm × 50 mm, 1.7 μm) that was operated at 40°C. The mobile phase consisted of Fisher Optima LC-MS grade CH_3_CN–H_2_O (both acidified with 0.1% formic acid). The gradient began at 15% CH_3_CN and linearly increased to 100% CH_3_CN over 8 min. It was held at 100% CH_3_CN for 1.5 min before returning to starting conditions to re-equilibrate (Supplementary Figure S4).

### Metabolomics Analyses

Principal component analysis (PCA) was conducted on the LC-MS data obtained for the large-scale fermentations of the mono and co-cultures. Untargeted LC-MS datasets for each sample were individually aligned, filtered, and analyzed using MZmine 2.20 software^1^ (Pluskal et al., 2010). Peak detection was achieved using the following parameters: noise level (absolute value), 1 × 10^6^; minimum peak duration, 0.05 min; *m/z* variation tolerance, 0.05; and *m/z* intensity variation, 20%. Peak list filtering and retention time (RT) alignment algorithms were used to refine peak detection. The join algorithm integrated all sample profiles into a data matrix using the following parameters: *m/z* and RT balance set at 10.0 each, *m/z* tolerance set at 0.001, and RT tolerance set at 0.5 min. The resulting data matrix was exported to Excel (Microsoft) for analysis as a set of *m/z* – RT pairs with individual peak areas detected in quadruplicate analyses. Samples that did not possess detectable quantities of a given marker ion were assigned a peak area of zero to maintain the same number of variables for all sample sets. Ions that did not elute between 2 and 8 min and/or had an *m/z* ratio less than 200 or greater than 800 were removed from analysis. Final chemometric analysis, including hierarchical cluster analysis and data filtering (Caesar et al., 2018) and PCA was conducted using Sirius version 10.0 (Pattern Recognition Systems AS) (Kvalheim et al., 2011). The PCA scores and loadings plots were generated using data from five individual biological replicates of the large scale fermentations. Each biological replicate was plotted using averaged peak areas obtained across four replicate injections (technical replicates). The same number of replicate analyses were used for each monoculture and the co-culture.

### Identifying Secondary Metabolite Genes in *A. fischeri* and *X. cubensis*

To identify which strains likely produced the secondary metabolites in co-culture we utilized the genome of *A. fischeri* and *Rosellinia necatrix*, a close evolutionary relative of *X. cubensis* (Hsieh et al., 2010). Individual proteins from the *Aspergillus clavatus* cytochalasin E cluster were used as web-based, blastp (Altschul et al., 1990) queries (accessed on 8-22-18) against the entire *X. cubensis* or *A. fischeri* proteomes found on the non-redundant protein sequences databases of NCBI. A Reciprocal Best BLAST approach (Salichos and Rokas, 2011) was performed to infer orthology between cytochalasin cluster proteins and blast hits found in *A. fischeri* and *X. cubensis*.

## Results

### Co-culture Analysis of the Junction Between *A. fischeri* and *X. cubensis* via Droplet Probe Reveals Varied Chemical Diversity Not Present in Either Monoculture

**FIGURE 3:**
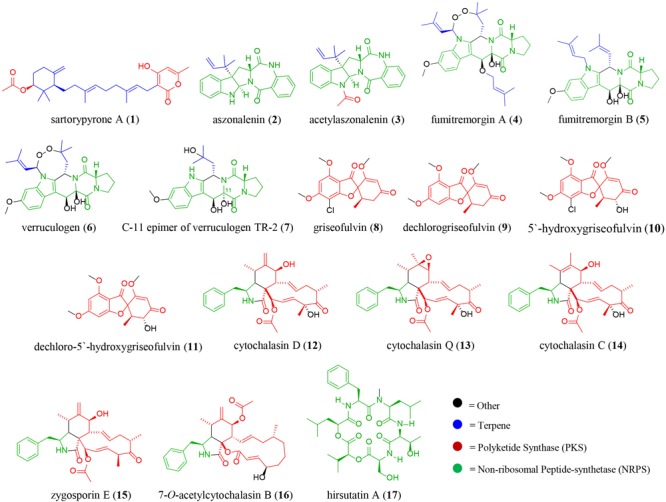
The diversity of secondary metabolites isolated from the co-culture of wild type *Aspergillus fischeri* (NRRL 181) and *Xylaria cubensis* (G536). The color of the structure indicates the different biosynthetic class of the secondary metabolites; blue, terpene; red, polyketide; green, non-ribosomal peptide, and black, other biosynthetic pathways.

**FIGURE 4:**
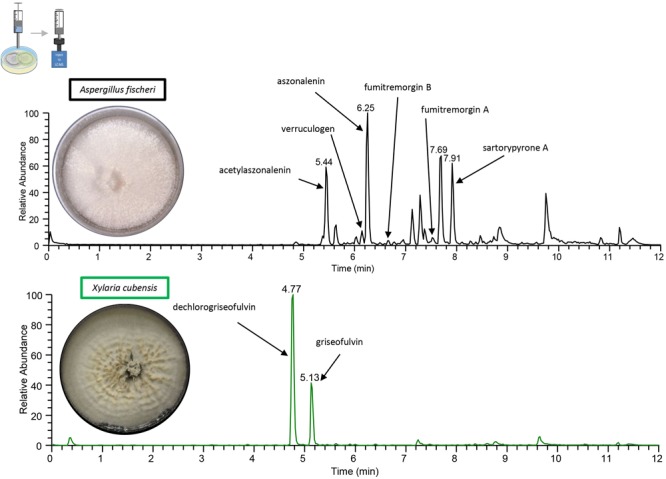
The chromatograms from the droplet probe analysis of the monocultures, as taken at the mycelial edge. Wild type *A. fischeri* (**top**; black) indicates the presence of sartorypyrone A (**1**), aszonalenin (**2**), acetylaszonalenin (**3**), fumitremorgin A (**4**), fumitremorgin B (**5**), and verruculogen (**6**). *X. cubensis* (**bottom**; green) shows dechlorogriseofulvin (**9**) and griseofulvin (**8**), which are analogues that have fungistatic activity. Only the isolated and fully characterized peaks are annotated.

To obtain baseline data, the monoculture of *X. cubensis* was sampled by droplet probe, and the LC-MS base peak chromatogram only showed two peaks, which corresponded to griseofulvin (**8**) and dechlorogriseofulvin (**9**) as determined by comparison of accurate mass and RT of a standard (El-Elimat et al., 2013; Paguigan et al., 2017). Griseofulvin (**8**) and dechlorogriseofulvin (**9**) were present in multiple spots across the mycelium, with both more concentrated at the mycelial edge. The monoculture of wild type *A. fischeri* was analyzed in an identical manner, and based on the same data analyses, sartorypyrone A (**1**) (Supplementary Figure S5), aszonalenin (**2**) (Supplementary Figure S6), acetylaszonalenin (**3**) (Supplementary Figure S7), fumitremorgin A (**4**) (Supplementary Figure S8), fumitremorgin B (**5**) (Supplementary Figure S9), verruculogen (**6**) (Supplementary Figure S10), and C-11 epimer of verruculogen TR-2 (**7**) (Supplementary Figure S11) were present (Figure 3, 4) (Mead et al., 2019).

### *In situ* Co-culture Analysis of *A. fischeri* and *X. cubensis* via Droplet Probe Reveals Varied Chemical Diversity at the Junction Between the Two Fungi Not Present in the Monoculture Experiments

**FIGURE 5:**
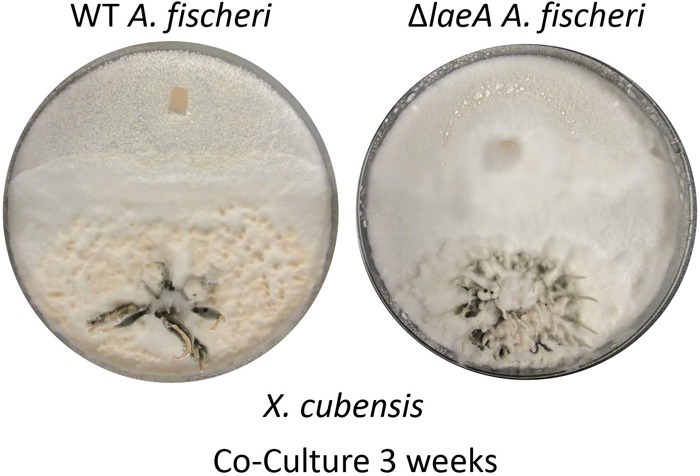
Wild type *A. fischeri* can form a deadlock while Δ*laeA A. fischeri* gets displaced by *X. cubensis* during the co-culture. The left image shows the growths of wild type *A. fischeri* and the right image shows the growths of Δ*laeA A. fischeri* both with *X. cubensis* over a 3-week timeline.

**FIGURE 6:**
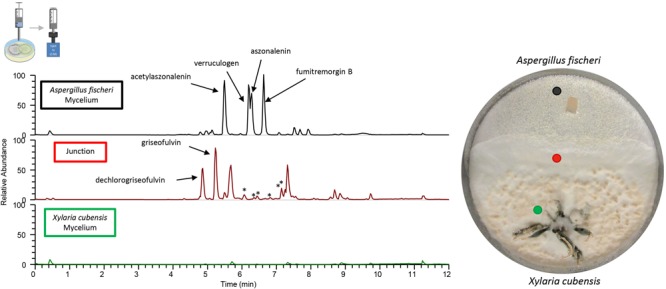
Co-culture produces greater diversity of secondary metabolites over that of the monocultures. The chromatograms from the droplet probe analysis of the co-cultures; only three out of the eight spots examined are shown here, with the corresponding colored dot indicating the location at which the mycelium was sampled. Wild type *A. fischeri* **(Top)** showed the presence of the secondary metabolites as seen in monoculture (Figure 4); however, there were differences in the relative abundance of the mycotoxins such as verruculogen and fumitremorgin B during their growth in co-culture. The junction **(middle)** where both fungal mycelia interact revealed the presence of griseofulvin and dechlorogriseofulvin, which were not present anywhere else in the co-culture. It also indicated the presence of secondary metabolites that were not present in the monoculture growths of either of the two fungi (asterisks). *X. cubensis* **(bottom)** did not show the presence of the two previously present secondary metabolites (griseofulvin and dechlorogriseofulvin), nor did it show the presence of any secondary metabolites.

A deadlock was formed in the *A. fischeri* and *X. cubensis* co-culture (Figure 5). The two fungi grew toward the middle of the plate, and at the junction where both fungal mycelia overlap, neither of the two was able to grow further (Supplementary Figure S2). The LC-MS profile showed distinct peaks (i.e., the presence of multiple ions) in co-culture (Figure 6), some of which were absent from the monocultures. Griseofulvin (**8**) and dechlorogriseofulvin (**9**) were not detected in the region of the co-culture where only *X. cubensis* mycelia were growing, however, both of these compounds were detected at the junction. Alternately, the monoculture had more accumulation of griseofulvin (**8**) and dechlorogriseofulvin (**9**) at the colony edge, but these compounds were present throughout the mycelia. The co-culture of wild type *A. fischeri* had the same secondary metabolites present as the monoculture. However, secondary metabolite production was altered during co-culturing (Figure 6). For example, verruculogen (**6**) and fumitremorgin B (**5**) showed an average increase in production of about 2 and 2.5 orders of magnitude, respectively, relative to their production in monoculture across three biological replicates *in situ*.

### Large-Scale Fermentation of Co-culture Indicated the Activation of Previously Silent Secondary Metabolites

**FIGURE 7:**
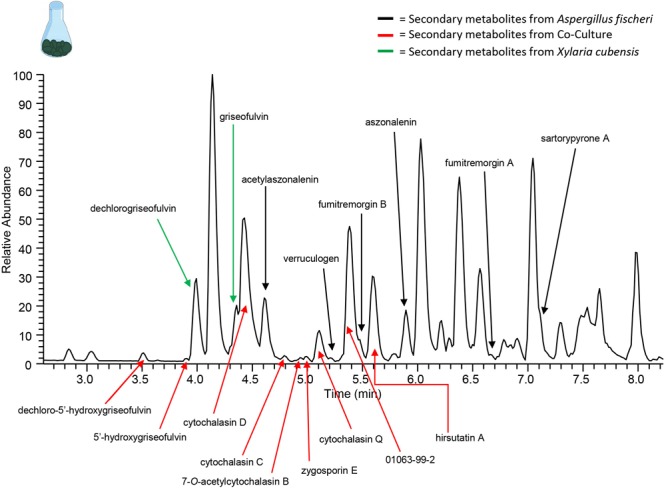
Increase in chemical diversity in the co-culture caused the characterization of secondary metabolite not seen in monoculture. The chromatogram produced from the large-scale extraction of mixed co-culture. Black arrows indicate the secondary metabolites from the monoculture of wild type *A. fischeri*; green arrows indicate the secondary metabolites from the monoculture of *X. cubensis*; and red arrows indicate the secondary metabolites isolated from the co-culture of wild type *A. fischeri* and *X. cubensis*.

To isolate and elucidate the structures of newly found secondary metabolite peaks in the junction between the two fungal species in the co-culture experiment, a large-scale fermentation of *X. cubensis* and wild type *A. fischeri* as a mixed co-culture on solid media was executed. The fungal growth was extracted and purified to isolate compounds that were characterized. In the co-culture griseofulvin (**8**) and dechlorogriseofulvin (**9**) (Paguigan et al., 2017), which were present in the monoculture, were isolated, as were the two analogs 5′-hydroxygriseofulvin (**10**) and dechloro-5′-hydroxygriseofulvin (**11**) (Paguigan et al., 2017), which were not observed in monoculture (Supplementary Figures S12–S15). The mycotoxins cytochalasin D (**12**) (Jikai et al., 2002), cytochalasin Q (**13**) (El-Elimat et al., 2013), cytochalasin C (**14**), zygosporin E (**15**) (Vedejs et al., 1988; Edwards et al., 1989), and 7-*O*-acetylcytochalasin B (**16**) (Capasso et al., 1991), (Figure 7) were observed in monoculture, albeit at extremely low concentrations that did not afford structural characterization. However, under co-culture conditions, they increased in abundance to an extent that afforded isolation and full structural characterization (Supplementary Figures S16–S19). Finally, hirsutatin A (**17**) (Isaka et al., 2005), which was not detected in the monoculture, was isolated and characterized in the large-scale co-culture (Supplementary Figure S20).

### Metabolomics Analyses Shows Different Chemical Entities in the Monoculture versus Co-culture

**FIGURE 8:**
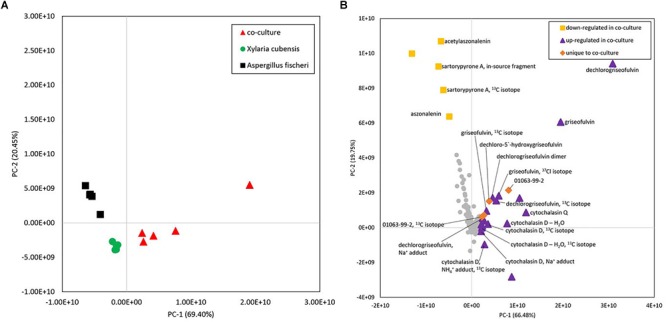
**(A)** Principal component analysis (PCA) scores plot of fungal samples shows distinct differences between the co-culture and monocultures. Five biological replicates of each sample type are plotted using peak area data for each sample that was an average of four injections (four technical replicates). Distinct clusters were observed between *X. cubensis, A. fischeri*, and co-cultured organisms. **(B)** Loadings plot from untargeted mass spectrometry based PCA of fungal samples shows the presence of unique and upregulated secondary metabolites in the co-culture. Metabolites with positive values along the horizontal axis were more heavily represented in co-cultures than monocultures or were unique to the co-culture entirely. Yellow squares, compounds that were detected at a lower abundance in the co-culture than in the monoculture of *Aspergillus fischeri.* Purple triangles, compounds that showed increased production in the co-culture and were produced by one or both fungi (Supplementary Table S1). Orange diamonds, compounds that were only detected in co-culture samples. Gray circles, compounds that do not contribute to the difference between the cultures. Metabolites were identified following isolation using a combination of mass spectral and NMR data. In cases where compounds were of too low abundance to be isolated, comparisons were made against the literature using *m/z* values from high-resolution mass spectrometry data. Identifications based on mass alone, without purification, are tentative.

Comparison of metabolite profiles is often achieved using multivariate statistical modeling protocols such as PCA (Ku et al., 2009). To determine the degree of difference between the metabolite profiles observed during the large-scale mono or co-culture, PCA scores and loadings plots were generated (Figure 8A,B). Untargeted metabolomics analyses of fungal samples using LC-MS yielded 2111 features (unique *m/z*-RT pairs) for 60 samples (five biological replicates of *X. cubensis, A. fischeri*, and the co-culture of the two fungal organisms, all analyzed in quadruplicate). Examination of these plots illustrated that clusters of *X. cubensis, A. fischeri*, and the co-culture of the two organisms were clustered distinctly in the PCA plot, which accounted for 89.9% of the variability among samples (PC1 = 69.4%, PC2 = 20.5%) (Figure 8A).

To evaluate which compounds contributed to the separation between groups, the PCA loadings plot was inspected (Figure 8B) using averaged data from both biological and technical replicates. Inspection of principal component loadings enables identification of variables that are responsible for the observed groupings in the PCA scores plot; the more a given variable diverges from other variables in the loadings plot, the more it contributes to the separation between groups in the scores plot. The two components utilized for this loadings plot explained 86.2% of the variability among samples (PC1 = 66.5%, PC2 = 19.8%). In this plot, compounds were identified as those that were (1) more abundant in the co-culture, (2) less abundant in the co-culture, and (3) unique to the co-culture (Supplementary Table S1). Metabolites with positive values along the *x*-axis, including griseofulvin analogs, cytochalasins, and a compound with a novel chemical scaffold (sample ID 01063-99-2) were detected in greater abundance in co-culture than in monocultures (purple triangles), or were completely unique to the co-culture (orange diamonds), and are responsible for the shift observed of the co-culture samples in the PCA scores plot.

### Genome Mining Implicates *X. cubensis* as the Species Producing Cytochalasin During the Co-culture

To determine whether *A. fischeri* or *X. cubensis* produced the cytochalasin congeners isolated from the co-culture, a combination of genome mining and mass spectrometry was used. Cytochalasins are a wide structural class of mycotoxins and are very common in Xylariales species (Chinworrungsee et al., 2001; Chen et al., 2011; Wei et al., 2015; Sharma et al., 2018). To attempt to determine which organism was producing cytochalasin D during the co-culture experiment, we searched for genes orthologous to a previously described biosynthetic gene cluster responsible for cytochalasin E production in *Aspergillus clavatus* (Qiao et al., 2011). A putative cytochalasin cluster was not found in *A. fischeri*, and only 4 out of 8 individual cytochalasin genes were identified in the genome and they were unclustered (Supplementary Table S2). Potential orthologs for the Non-ribosomal Peptide Synthase-Polyketide Synthase hybrid and enoyl reductase genes were found in a biosynthetic gene cluster in *A. fischeri* that likely produces the secondary metabolite aspyridone (Mead et al., 2019). Sequencing of the genome of *X. cubensis* has not been reported, and the closest evolutionary relative that has a sequenced genome is *Rosellinia necatrix* (Hsieh et al., 2010; Sica et al., 2016c). Upon analysis of the *R. necatrix* genome, a complete cytochalasin gene cluster was found (Supplementary Table S2) and led us to hypothesize that *X. cubensis* was responsible for biosynthesizing the cytochalasins isolated from the batch co-culture. Furthermore, un-isolatable amounts of the cytochalasins were detected by mass spectrometry in the mono-culture of *X. cubensis*, thus supporting our hypothesis that the cytochalasins were most likely biosynthesized by *X. cubensis* during co-culture.

### *In situ* Analysis of a Co-culture Between Δ*laeA A. fischeri* and *X. cubensis* Reveals the Ecological Importance of Secondary Metabolites in Interspecies Chemical Interactions

A less diverse secondary metabolite profile was observed in Δ*laeA A. fischeri* (Supplementary Figure S1) compared to the wild type *A. fischeri*, while the growth rates between the strains was equivalent (Mead et al., 2019). Mycelial displacement was observed when Δ*laeA A. fischeri* was grown with *X. cubensis*. Once the junction was formed (about 2 weeks), *X. cubensis* continued to grow and took up more territory from the Δ*laeA A. fischeri*. After 3 weeks the mycelium of Δ*laeA A. fischeri* had been covered by *X. cubensis* (Figure 5).

## Discussion

In this study, we performed the chemical analysis of two fungi, *Aspergillus fischeri* and *Xylaria cubensis*, under both monoculture and co-culture conditions to characterize the secondary metabolites produced by these fungi using both *in situ* analysis with droplet probe as well as large-scale fermentation (Figure 1). Specifically, we found that the secondary metabolites identified by comparing the analytical profiles in monoculture were different than those observed from co-culture *in situ* (Figure 4, 6). In co-culture, the signals for secondary metabolites present at the junction, or the region of conflict zone (Hautbergue et al., 2018), appeared to be unique and were formed as a result of the interspecies cross talk between the two fungi (Figure 6). We also found that when wild type *A. fischeri* was co-cultured with *Xylaria cubensis*, a junction or conflict zone was formed. However, when Δ*laeA A. fischeri* was co-cultured with *X. cubensis*, the conflict zone was disturbed and Δ*laeA A. fischeri* was displaced by *X. cubensis* (Figure 5). When *X. cubensis* and wild type *A. fischeri* were co-cultured in large-scale, so as to isolate and characterize the compounds, we showed an increase in chemical diversity, including a putative novel scaffold, suggesting the co-culture stimulated cryptic biosynthesis in one or both fungi (Figure 7).

*In situ* analysis of co-culture between wild type *A. fischeri* and *X. cubensis* reveals that chemistry not previously observed in monocultures was produced at the junction or conflict zone in interspecies chemical interactions. Most studies of fungi-fungi co-culture or fungi-bacteria co-culture demonstrate that when two microbes are co-cultivated, new chemical compounds are biosynthesized by one or both microbes as a result of some interspecies interaction or interspecies cross talk (Marmann et al., 2014). Using droplet probe, we observed new chemical profiles at the junction between the two-competing species (Figure 6). A similar result was found in a co-culture study between *Aspergillus nidulans* with *Streptomyces hygroscopicus* (Schroeckh et al., 2009), which reported the physical interaction and close contact between *S. hygroscopicus* and *A. nidulans* stimulated the production of aromatic polyketides. Another recent study, which performed *in situ* mapping using MALDI-imaging-HRMS, demonstrated the increased production of prodigiosin produced by endophytic *Serratia marcescens* when grown in co-culture with endophytic fungi (Eckelmann et al., 2018), where higher amounts of prodigiosin were produced at the contact site between the fungal and bacterial cultures. Thus, by sampling fungi and their surrounding environment, *in situ* methods have the ability to map the location of fungal metabolites. The ability to map chemical diversity of secondary metabolites *in situ* provides an ability to probe biological questions directly, such as why the fungus produces such compounds and how they are spatially distributed. Determining whether a compound is produced for defense, communication, attraction, or other purposes, can be explored via these mapping experiments (Sica et al., 2014, 2016c).

*In situ* visualization of griseofulvin production in monoculture versus co-culture was different, due to the differential accumulation of griseofulvin (**8**), suggesting how antagonistic or combative species use allelochemicals for attack on other microbes to break through the conflict zone. Griseofulvin is an antifungal compound that can be classified as an allelochemical (allomone), which is implicated in interspecies interactions that can benefit the producing organism, but not the receiving one (Drijfhout, 2001; Sica et al., 2016c; Paguigan et al., 2017). The question of how fungi protect themselves from such armaments is often pondered. They seem to have at least three specific mechanisms to do so, including the up regulation of efflux transporters, detoxifying enzymes, and duplicate copies of the target protein (Keller, 2015, 2018; Gilchrist et al., 2018).

Furthermore, our study provides a striking visualization of how fungi use secondary metabolites during interspecies interactions. We demonstrate that when *X. cubensis* is grown in monoculture and sampled via droplet probe, griseofulvin (**8**) and dechlorogriseofulvin (**9**) are present in the mycelium but mostly toward the outer edge of the growing colony (Figure 4). This was observed in three biological replicates *in situ*, indicating that *X. cubensis* may be marshaling these fungistatic allelochemicals to the front line of the “battlefield.” Similarly, previous literature also reports that when *X. cubensis* is grown in co-culture with *Penicillium restrictum*, that the majority of griseofulvin was exuded to the colony edge (Sica et al., 2016c), thus suggesting it makes the antifungal compound but does not keep it inside its own mycelium, perhaps protecting itself via enhancement of efflux (Gilchrist et al., 2018; Keller, 2018). In the present study, we see that in fact there is no trace of antifungal compound in the mycelium of *X. cubensis* in co-culture (Figure 6). Rather, we observe that several fungistatic antifungal compounds (griseofulvin (**8**), dechlorogriseofulvin (**9**), and two analogs 5′-hydroxygriseofulvin (**10**) and dechloro-5′-hydroxygriseofulvin (**11**)) are sent out toward the junction where the two species have formed a deadlock or conflict zone (Figure 5, 6). Thus, the *in situ* experiments demonstrate how *X. cubensis* is using its secondary metabolite arsenal to stop the growth of wild type *A. fischeri*.

Overproduction of griseofulvin has also been reported in guttates from *X. cubensis*, when it was grown in media enriched with the antifungal compound amphotericin B (Caraballo-Rodríguez et al., 2017). Our spatial study of griseofulvin and its analogs using droplet probe provides experimental evidence that in more complex environments, such as co-cultures, the location and the amounts of key secondary metabolites (i.e., griseofulvin and analogs) are both up regulated as well as outwardly extruded toward the competing fungal species. In a previous study, we also found that when *Coniolariella* spp., which is known to produce the herbicidal compound mevalocidin (Gerwick et al., 2013), were examined by droplet probe, the secondary metabolites were concentrated in the guttates and in the surrounding media (Sica et al., 2016b). These data confirm our observation in the present study that antagonistic compounds are extruded outwardly toward the receiving organism for maximum benefit to the producing organism. Together, our results suggest that both the amounts and spatial distributions of secondary metabolites can vary during interspecies interactions.

When wild type *A. fischeri* and *X. cubensis* were grown together, a deadlock was formed (Figure 5), where neither fungus was able to capture territory from the other (Hiscox et al., 2018). Even though *X. cubensis* was able to biosynthesize secondary metabolites that stunt the growth of the competing fungus, *A. fischeri* was most likely able to compete due to the presence of its secondary metabolite arsenal, such as the fumitremorgin class of alkaloids (**4**-**7**). Droplet probe analysis of the co-culture of wild type *A. fischeri* and *X. cubensis in situ* suggested that no new secondary metabolites were biosynthesized by wild type *A. fischeri* (Figure 6). However, in the three spatial locations sampled in the co-culture where there was only *A. fischeri* mycelium, there was a change in the relative abundance of the mycotoxins produced. For example, the relative abundance of the mycotoxins verruculogen (**6**) (on average 99-fold) and fumitremorgin B (**5**) (on average 156-fold) increased in co-culture versus monoculture, as seen by the area under the curve (Supplementary Table S3). This was observed across three separate biological replicates *in situ*, suggesting that *A. fischeri* increased the abundance of its mycotoxin biosynthesis to better compete with *X. cubensis* (Reverberi et al., 2010; Ehrlich et al., 2011).

Manipulating global transcriptional regulators may shed light on the biosynthesis of secondary metabolites in fungi (Skellam, 2018). The deletion mutant of *laeA* provides support to the notion that secondary metabolites are akin to a fungal arsenal (Rokas et al., 2018); if they are no longer available to the fungus, it loses the space, and eventually the battle, during interspecies interactions. *LaeA* is an important regulator of secondary metabolism in *Aspergillus* spp. (Bok and Keller, 2004; Sarikaya-Bayram et al., 2015). We confirmed this observation in *A. fischeri*, when we studied the chemical profiles of Δ*laeA A. fischeri* (Mead et al., 2019). Our results found that the majority of the secondary metabolites produced by the wild type strain were produced at a lower abundance in the Δ*laeA A. fischeri* (Mead et al., 2019). Based on those findings we hypothesized that because the Δ*laeA* strain has a perturbed secondary metabolite arsenal, it would be unable to compete with griseofulvin producing *X. cubensis.* When the Δ*laeA* strain was co-cultured with *X. cubensis*, the conflict zone was disturbed, and Δ*laeA A. fischeri* was displaced by outward production of griseofulvin and its analogs by *X. cubensis* (Figure 5). Our study thus provides visual evidence using *in situ* mapping that fungi utilize secondary metabolites in order to compete ecologically with other microbes for nutrition and space. We hypothesize here that if the chemical diversity of secondary metabolites is perturbed (*ΔlaeA A. fischeri*), it could lead to loss of small molecules, the loss of territory, and defeat in the fungal battlefield during interspecies interactions (O’Brien and Wright, 2011). Further studies using genetic knock outs are, however, necessary to provide further support of our hypothesis.

Previously silenced secondary metabolites are expressed in co-culture during stressful conditions, likely due to the activation of silent genes (Pettit, 2009; Schroeckh et al., 2009; Marmann et al., 2014; Li and Lou, 2017; Xu et al., 2018). Because Petri plates with nutrient agar produce a low yield, particularly when only a small portion of the plate has the chemistry of interest (i.e., the junction), a scale up study was conducted to enhance the amounts of secondary metabolites. The scale up allowed for characterization of the newly activated secondary metabolites (Figure 7), as well as the secondary metabolites previously present (Figure 4). It confirmed the presence of compounds **1 – 7** from *A. fischeri*, as well as compound **8** and **9** in *X. cubensis*. It also showed the activation of griseofulvin analogs (compounds **10** and **11**), all of which have fungistatic properties. Similarly, the biosynthesis of cytochalasin mycotoxins were activated in co-culture conditions, and hirsutatin A (**17**) was also isolated from the mixed co-culture (Isaka et al., 2005). This compound has only been reported from an insect pathogenic fungus *Hirsutella nivea* (Hypocreales, Ascomycota), but never from *A. fischeri* nor *X. cubensis.* There is no biosynthetic gene cluster linked to this metabolite, but with a trace amount being detectable in the monoculture of *X. cubensis*, we hypothesize that it may have biosynthesized this secondary metabolite. The activation of these defensive secondary metabolites during co-culturing was most likely due to the signaling and threat assessment that occurred between the two fungi, suggesting that hirsutatin A (**17**) provides *X. cubensis* with some sort of competitive advantage.

Among the new signals present in the chromatographic profile of the large-scale co-culture, we were pleased to find an unknown peak (01063-99-2; Figure 7), which upon chemical characterization revealed a novel chemical scaffold suggesting that co-culturing of the two cultures in large-scale resulted in the stimulation of cryptic biosynthetic gene clusters in one or both fungi. The complete chemical characterization and identification of the producer strain of the unknown peak is currently ongoing. Numerous previous studies have found new chemistry in large-scale co-culture fermentations (Nonaka et al., 2011; Adnani et al., 2017; Shang et al., 2017; Wang W. et al., 2018).

## Conclusion

Fungal interactions are fascinating, but how these chemical ecology interactions occur in nature is poorly understood. This is mainly due to the difficulty of measuring secondary metabolites *in situ* during fungal interactions, which hinders our understanding of what happens when two fungi interact. The droplet probe technique helped gain insight into the ecology of interspecies interactions and spatial distribution of the secondary metabolites *in situ*, information that is typically lost through a traditional extraction protocol. Our data revealed that while fungi are growing in a more complex environment (co-culture), they respond differently to interspecies interaction and alter the distribution and production of key secondary metabolites accordingly. Based on the results presented herein, we hypothesize that *ΔlaeA A. fischeri* co-culture experiments demonstrate that secondary metabolites may provide a competitive advantage to the producing fungi, and that such metabolites could play an important role in shaping interspecies chemical interactions. A total of 18 secondary metabolites were biosynthesized in large-scale co-culture. Of those metabolites, two were characterized from the monoculture of *X. cubensis* and seven were characterized from the monoculture of *A. fischeri*. Thus, nine were characterized solely from the co-culture experiment, which included a putative novel scaffold that was biosynthesized only in co-culture. If we are to understand the full extent of fungal secondary metabolites in drug discovery, and the potential role of such metabolites in structuring fungal communities in nature, *in situ* information about species interactions for a wide variety of fungi is required.

## Author Contributions

SK carried out the mass spectrometry analysis and the chemical characterization of the secondary metabolites. She also helped with the general design of the experiments and wrote the manuscript. HR dealt with the mycology aspects of the science, and he also helped write the manuscript. AW carried out the purification of secondary metabolites under the guidance of SK. AL assisted with the mycology aspects under the guidance of HR. LC and NC carried out the metabolomics aspects and assisted with the writing of the manuscript. MM, JS, AR carried out the genetic aspects of this manuscript and MM and AR both helped in writing the manuscript. LR and GG prepared the knock-out strain. NO led the project, particularly the experimental design and writing of manuscript.

## Conflict of Interest Statement

The authors declare that the research was conducted in the absence of any commercial or financial relationships that could be construed as a potential conflict of interest.

## Footnotes

1 http://mzmine.sourceforge.net/
