# Supplementary material for: Mapping the Fungal Battlefield: Using in situ Chemistry and Deletion Mutants to Monitor Interspecific Chemical Interactions Between Fungi
Source: Front Microbiol. 2019 Feb 19;10:285. doi: 10.3389/fmicb.2019.00285 (PMC6389630; doi:10.3389/fmicb.2019.00285)
Supplement: Supplementary file 1 [file Data_Sheet_1.docx]

**Mapping the Fungal Battlefield: Using *in situ* Chemistry and Deletion Mutants to Monitor Interspecific Chemical Interactions between Fungi**

**Authors:** Sonja L. Knowles^1^, Huzefa A. Raja^1^, Allison J. Wright^1^, Ann Marie L. Lee^1^, Lindsay K. Caesar^1^, Nadja B. Cech^1^, Matthew E. Mead^2^, Jacob L. Steenwyk^2^, Laure N. A. Ries^3^, Gustavo H. Goldman^3^, Antonis Rokas^2^, Nicholas H. Oberlies^1*^

**Figure S1.** Wild type and Δ*laeA* *Aspergillus fischeri* and *Xylaria cubensis* grown on Petri plates

**Figure S2.** Co-culture growths

**Figure S3.** Base peak of all eight spots across the co-culture

**Figure S4.** Photodiode-Array (PDA) Detector Chromatogram of secondary metabolites

**Figure S5.** ^1^H NMR spectrum (700 MHz, Top) and ^13^C NMR spectrum (175 MHz, Bottom) both in CDCl_3_, of sartorypyrone A (**1**)

**Figure S6.** ^1^H NMR spectrum (400 MHz) in CDCl_3_, aszonalenin (**2**)

**Figure S7.** ^1^H NMR spectrum (500 MHz, Top) and ^13^C NMR spectrum (125 MHz, Bottom) both in CDCl_3_, of acetylaszonalenin (**3**)

**Figure S8.** ^1^H NMR spectrum (500 MHz, Top) and ^13^C NMR spectrum (125 MHz, Bottom) both in CDCl_3_, of fumitremorgin A (**4**)

**Figure S9.** ^1^H NMR spectrum (700 MHz, Top) and ^13^C NMR spectrum (175 MHz, Bottom) both in CDCl_3_, of fumitremorgin B (**5**)

**Figure S10.** ^1^H NMR spectrum (500 MHz, Top) and ^13^C NMR spectrum (125 MHz, Bottom) both in CDCl_3_, of verruculogen (**6**)

**Figure S11.** ^1^H NMR spectrum (700 MHz, Top) and ^13^C NMR spectrum (175 MHz, Bottom) both in CDCl_3_, of C-11 epimer of verruculogen TR-2 (**7**)

**Figure S12.** ^1^H NMR spectrum (400 MHz) in CDCl_3_, griseofulvin (**8**)

**Figure S13.** ^1^H NMR spectrum (400 MHz) in CDCl_3_, dechlorogriseofulvin (**9**)

**Figure S14.** ^1^H NMR spectrum (400 MHz) in CDCl_3_, 5`-hydroxygriseofulvin (**10**)

**Figure S15.** ^1^H NMR spectrum (400 MHz) in CDCl_3_, dechloro-5`-hydroxygriseofulvin (**11**)

**Figure S16.** ^1^H NMR spectrum (400 MHz, Top) and ^13^C NMR spectrum (100 MHz, Bottom) both in CDCl_3_, of cytochalasin D (**12**)

**Figure S17.** ^1^H NMR spectrum (400 MHz) in CDCl_3_, cytochalasin C (**14**)

**Figure S18.** ^1^H NMR spectrum (400 MHz) in CDCl_3_, zygosporin E (**15**)

**Figure S19.** ^1^H NMR spectrum (400 MHz) in CDCl_3_, 7-*O*-acetylcytochalasin B (**16**)

**Figure S20.** ^1^H NMR spectrum (400 MHz) in CDCl_3_, hirsutatin A (**17**)

**Table S1**. List of altered features in the co-culture of *X. cubensis* and *A. fischeri* analyzed by LC-MS

**Table S2.** Cytochalasin gene cluster analysis

**Table S3.** Biological replicates Area Under the Curve for average increase in mycotoxins

**Figure S1.** Wild type and Δ*laeA* *Aspergillus fischeri* and *Xylaria cubensis* grown on Petri plates

| 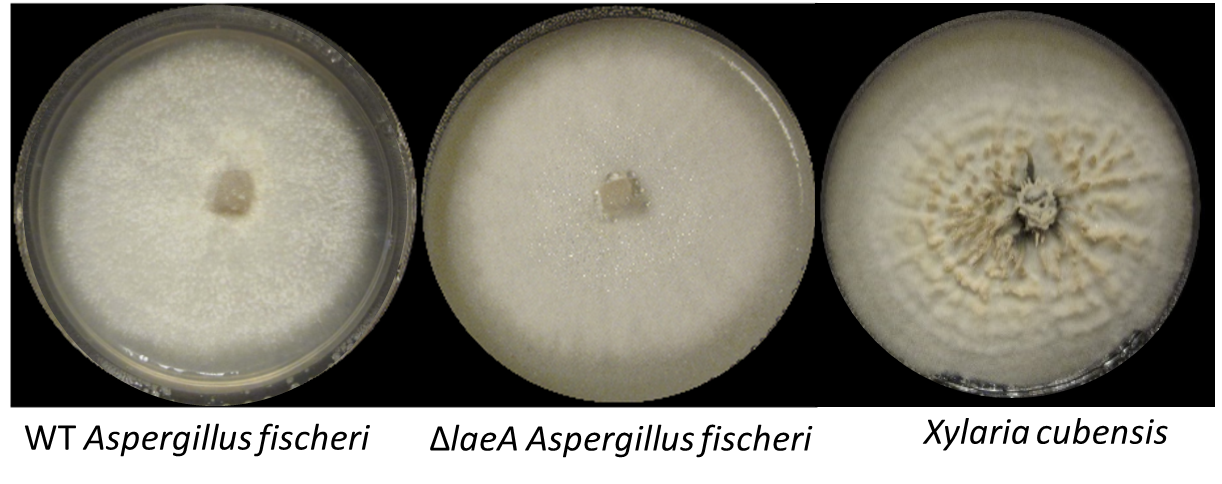 |
| --- |
| **A.** Wild type *Aspergillus fischeri* (NRRL 181); **B.** *ΔlaeA* *Aspergillus fischeri*; **C.** *Xylaria cubensis* (G536) grown on oatmeal agar (Difco). |

**Figure S2.** Co-culture growths

**Figure S3.** Base peak of all eight spots across the co-culture

A total of eight locations were sampled across the co-culture (three spots on *Aspergllus fischeri* mycelium, two spots at the junction, two spots on *Xylaria cubensis* mycelium, and one spot on *Xylaria cubensis* stroma). Spatial difference of secondary metabolites across the co-culture (Plate 1) is shown using stacked chromatograms.

**Figure S4.** Photodiode-Array (PDA) Detector Chromatogram of secondary metabolites


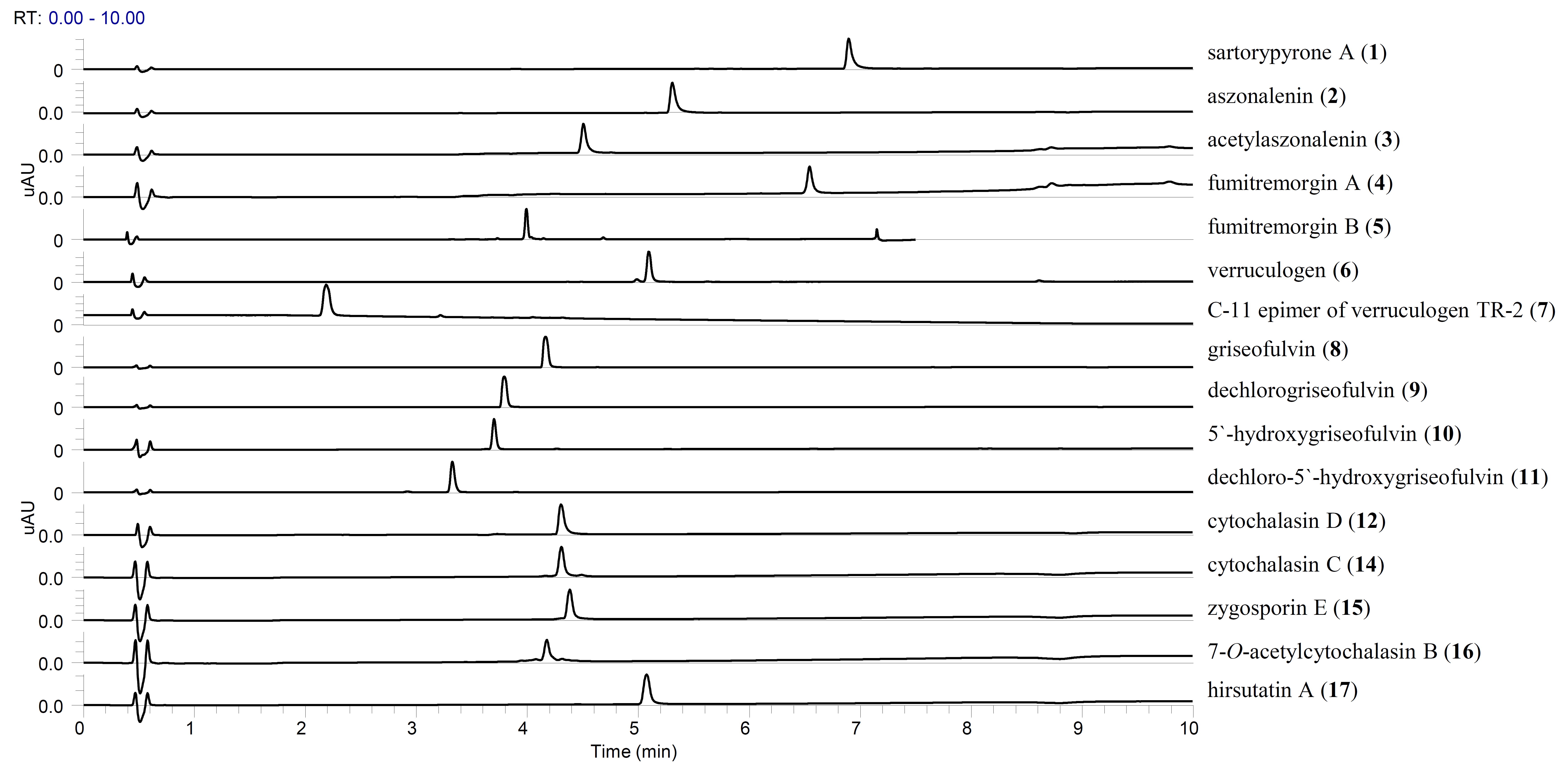


**Figure S5.** ^1^H NMR spectrum (700 MHz, Top) and ^13^C NMR spectrum (175 MHz, Bottom) both in CDCl_3_, of sartorypyrone A (**1**)

**Figure S6.** ^1^H NMR spectrum (400 MHz) in CDCl_3_, aszonalenin (**2**)

**Figure S7.** ^1^H NMR spectrum (500 MHz, Top) and ^13^C NMR spectrum (125 MHz, Bottom) both in CDCl_3_, of acetylaszonalenin (**3**)

**Figure S8.** ^1^H NMR spectrum (500 MHz, Top) and ^13^C NMR spectrum (125 MHz, Bottom) both in CDCl_3_, of fumitremorgin A (**4**)

**Figure S9.** ^1^H NMR spectrum (700 MHz, Top) and ^13^C NMR spectrum (175 MHz, Bottom) both in CDCl_3_, of fumitremorgin B (**5**)

**Figure S10.** ^1^H NMR spectrum (500 MHz, Top) and ^13^C NMR spectrum (125 MHz, Bottom) both in CDCl_3_, of verruculogen (**6**)

**Figure S11.** ^1^H NMR spectrum (700 MHz, Top) and ^13^C NMR spectrum (175 MHz, Bottom) both in CDCl_3_, of C-11 epimer of verruculogen TR-2 (**7**)

**Figure S12.** ^1^H NMR spectrum (400 MHz) in CDCl_3_, griseofulvin (**8**)

**Figure S13.** ^1^H NMR spectrum (400 MHz) in CDCl_3_, dechlorogriseofulvin (**9**)

**Figure S14.** ^1^H NMR spectrum (400 MHz) in CDCl_3_, 5`-hydroxygriseofulvin (**10**)

**Figure S15.** ^1^H NMR spectrum (400 MHz) in CDCl_3_, dechloro-5`-hydroxygriseofulvin (**11**)

**Figure S16.** ^1^H NMR spectrum (400 MHz, Top) and ^13^C NMR spectrum (100 MHz, Bottom) both in CDCl_3_, of cytochalasin D (**12**)

**Figure S17.** ^1^H NMR spectrum (400 MHz) in CDCl_3_, cytochalasin C (**14**)

**Figure S18.** ^1^H NMR spectrum (400 MHz) in CDCl_3_, zygosporin E (**15**)

**Figure S19.** ^1^H NMR spectrum (400 MHz) in CDCl_3_, 7-*O*-acetylcytochalasin B (**16**)

**Figure S20.** ^1^H NMR spectrum (400 MHz) in CDCl_3_, hirsutatin A (**17**)

**Table S1**. List of altered features in the co-culture of *X. cubensis* and *A. fischeri* analyzed by LC-MS

| **Compound** | **Ion/retention time^a^ (molecular formula, δ [ppm])** | **Adducts, fragments, and isotopes (molecular formula, δ [ppm])** | **Difference Among Samples** | **Produced by Samples** |
| --- | --- | --- | --- | --- |
| **Dechlorogriseofulvin** | 319.118 [M+H]^+^/3.928 (C_17_H_19_O_6_^+^, 0.501) | 320.120 [M+H]^+^, ^13^C isotope (C_27_H_19_O_6_^+^, 3.124)  341.099 [M+Na]^+^ ([C_27_H_18_O_6_ + Na]^+^, 2.932)  637.228 [2M+H]^+^, ([2C_27_H_18_O_6_ + H]^+^, 0.785) | Higher abundance in mixed co-culture | *X. cubensis*, co-culture |
| **Unknown Metabolite A** | 324.209 [M+H]^+^/6.932 (C_22_H_28_O_2_^+^, 0.215)^b^ |  | Higher abundance in mixed co-culture | *X. cubensis*, *A. fischeri* , co-culture |
| **Unknown Metabolite B** | 326.305 [M+H]^+^/7.502 (C_20_H_40_NO_2_^+^, 0.905) |  | Higher abundance in mixed co-culture | *X. cubensis*, *A. fischeri* , co-culture |
| **Dechloro-5’-hydroxygriseofulvin** | 335.113 [M+H]^+^/3.445 (C_17_H_19_O_7_^+^, 0.233) |  | Unique to mixed co-culture | Co-culture |
| **Griseofulvin** | 353.079 [M+H]^+^/4.269 (C_17_H_18_ClO_6_^+^, 0.538) | 354.081 [M+H]^+^, ^13^C isotope (C_17_H_18_ClO_6_^+^, 4.236)  355.075 [M+H]^+^, ^37^Cl isotope (C_17_H_18_ClO_6_^+^, 2.816) | Higher abundance in mixed co-culture | *X. cubensis*, co-culture |
| **Aszonalenin** | 374.186 [M+H]^+^/5.321 (C_23_H_24_N_3_O_2_^+^, 2.271) |  | Lower abundance in mixed co-culture | *A. fischeri* |
| **Sartorypyrone A** | 457.295 [M+H]^+^/7.054 (C_28_H_41_O_5_^+^, 1.159) | 458.298 [M+H]^+^, ^13^C isotope (C_28_H_41_O_5_^+^, 1.724)  397.274 [M+H-acetic acid]^+^ ([C_28_H_41_O_5_-C_2_H_4_O_2_]^+^, 0.680) | Lower abundance in mixed co-culture | *A. fischeri* |
| **01063-99-2** | 413.232 [M+H]^+^/5.308 (C_25_H_33_O_5_^+^, 1.936) | 414.235 [M+H]^+^, ^13^C isotope  (C_25_H_33_O_5_^+^, 2.414) | Unique to mixed co-culture | Co-culture |
| **Acetylaszonalenin** | 416.197 [M+H]^+^/4.552 (C_25_H_26_N_3_O_3_^+^, 1.009) |  | Lower abundance in mixed co-culture | Co-culture, *A. fischeri* |
| **Unknown Metabolite C** | 431.242 [M+H]^+^/5.306 (C_25_H_35_O_6_^+^, 2.226) |  | Unique to mixed co-culture | Co-culture |
| **Cytochalasin D** | 508.269 [M+H]^+^/4.433 (C_30_H_38_NO_6_^+^, 1.790) | 509.273 [M+H]^+^, ^13^C isotope (C_30_H_38_NO_6_^+^, 0.589)  490.258 [M+H-H_2_O]^+^ ([C_30_H_38_NO_6_-H_2_O]^+^, 2.754)  491.262 [M+H-H_2_O]^+^, ^13^C isotope  ([C_30_H_38_NO_6_-H_2_O]^+^, 1.486)  526.300 [M+NH_4_]^+^, ^13^C isotope ([C_30_H_37_NO_6_+NH_4_]^+^, 0.266)  530.251 [M+Na]^+^ ([C_30_H_37_NO_6_+Na]^+^, 1.622) | Higher abundance in mixed co-culture | *X. cubensis*, co-culture |
| **Cytochalasin Q^c^** | 508.269 [M+H]^+^/5.057 (C_30_H_38_NO_6_^+^, 1.790) |  | Higher abundance in mixed co-culture | *X. cubensis*, co-culture |
| **Unknown Metabolite D** | 508.270 [M+H]^+^/4.686 (C_30_H_38_NO_6_^+^, 0.177) | 525.296 [M+NH_4_]^+^, ([C_30_H_37_NO_6_+NH_4_]^+^, 0.876) | Higher abundance in mixed co-culture | *X. cubensis*, co-culture |
| **Unknown Metabolite E** | 496.339 [M+H]^+^/6.312 (C_28_H_48_O_7_^+^, 0.403) |  | Lower abundance in mixed co-culture | *A. fischeri* |
| **Hirsutatin A** | 677.374 [M+H]^+^/5.514 (C_34_H_53­_N_4_O_10_^+^, 0.0) | 694.407 [M+NH_4_]^+^ | Higher abundance in mixed co-culture | *X. cubensis*, co-culture |

List of altered features in the co-culture of *X. cubensis* and *A. fischeri* analyzed by LC-MS.

^a^ RT in minutes

^b^ based on isotope patterns, adduct formation, and accurate mass, most likely a cytochalasin

^c^ cytochalasin Q from in house dereplication database, which analyzes molecular weight, retention time, UV absorbance, and MS2 data.

**Table S2.** Putative orthologs of the cytochalasin gene cluster found in *X. cubensis* and *A. fischeri*.

| *A. clavatus* Gene ID | Putative function | *R. necatrix* Protein ID | % Query Coverage | % Identity | E-value | RBBH? |
| --- | --- | --- | --- | --- | --- | --- |
| ACLA_078640 | Zn2-Cys6 Binuclear Cluster Transcription Factor | GAP90887.2 | 98 | 38 | 1.00E-62 | YES |
| ACLA_078650 | Baeyer-Villiger monooxygenase | GAP90886.1 | 94 | 69 | 0 | YES |
| ACLA_078660 | NRPS-PKS Hybrid | GAP90879.1 | 99 | 64 | 0 | YES |
| ACLA_078670 | p450 | GAP90880.1 | 97 | 73 | 0 | YES |
| ACLA_078680 | a/b Hydrolase | GAP90882.1 | 100 | 74 | 0 | YES |
| ACLA_078690 | Hypothetical Protein | GAP90883.1 | 99 | 76 | 0 | YES |
| ACLA_078700 | Enoyl Reductase | GAP90884.1 | 97 | 74 | 0 | YES |
| ACLA_078710 | p450 | GAW26913.1 | 71 | 58 | 1.00E-157 | YES |

**Table S3.** Biological replicates Area Under the Curve for average increase in mycotoxins during the co-culture

| Sample Location | Verruculogen AUC | Fumitremorgin B AUC |
| --- | --- | --- |
| Monoculture *A. fischeri* plate 1 | 4802578 | 3500315 |
| Monoculture *A. fischeri* plate 2 | 1244114 | 364643 |
|  |  |  |
| Average monoculture | 3023346 | 1932479 |
|  |  |  |
| Co-culture *A. fischeri* mycelium  plate 1 and spot 1 | 271914463 | 96673066 |
| Co-culture *A. fischeri* mycelium  plate 1 and spot 2 | 366115822 | 68873847 |
| Co-culture *A. fischeri* mycelium  plate 1 and spot 3 | 71382535 | 26574515 |
| Co-culture *A. fischeri* mycelium  plate 2 and spot 1 | 1524267537 | 1693328571 |
| Co-culture *A. fischeri* mycelium  plate 2 and spot 2 | 469851785 | 309567783 |
| Co-culture *A. fischeri* mycelium  plate 2 and spot 3 | 345340713 | 64012614 |
| Co-culture *A. fischeri* mycelium  plate 3 and spot 1 | 120837062 | 56565051 |
| Co-culture *A. fischeri* mycelium  plate 3 and spot 2 | 590479791 | 78819794 |
|  |  |  |
| Average Co-culture | 470023714 | 299301905 |
|  |  |  |
| Co-culture fold increase over monoculture | 155.5 | 99.0 |
